# Cardiac Magnetic Resonance Identifies Responders to Cardiac Resynchronization Therapy with an Assessment of Septal Scar and Left Ventricular Dyssynchrony

**DOI:** 10.3390/jcm12227182

**Published:** 2023-11-20

**Authors:** Camilla Kjellstad Larsen, Otto A. Smiseth, Jürgen Duchenne, Elena Galli, John Moene Aalen, Mathieu Lederlin, Jan Bogaert, Erik Kongsgaard, Cecilia Linde, Martin Penicka, Erwan Donal, Jens-Uwe Voigt, Einar Hopp

**Affiliations:** 1 Institute for Surgical Research and Department of Cardiology, Oslo University Hospital, 0027 Oslo, Norway; camillakjellstadlarsen@gmail.com (C.K.L.); otto.smiseth@gmail.com (O.A.S.); john.aalen@gmail.com (J.M.A.); 2 Institute of Clinical Medicine, University of Oslo, 0316 Oslo, Norway; 3 Department of Cardiovascular Diseases, University Hospitals Leuven, 3000 Leuven, Belgiumjens-uwe.voigt@uzleuven.be (J.-U.V.); 4 Department of Cardiovascular Sciences, KU Leuven—University of Leuven, 3000 Leuven, Belgium; 5 Department of Cardiology, University Hospital of Rennes, 35000 Rennes, France; elena.galli@chu-rennes.fr (E.G.); erwan.donal@chu-rennes.fr (E.D.); 6 Department of Radiology, University Hospital of Rennes, 35000 Rennes, France; mathieu.lederlin@chu-rennes.fr; 7 Department of Radiology, University Hospitals Leuven, 3000 Leuven, Belgium; jan.bogaert@uzleuven.be; 8 Department of Cardiology, Oslo University Hospital, 0027 Oslo, Norway; ekongsga@ous-hf.no; 9 Department of Cardiology, Karolinska University Hospital, 171 64 Solna, Sweden; cecilia.linde@ki.se; 10 Cardiovascular Center, OLV Clinic, 9300 Aalst, Belgium; martin.penicka@olvz-aalst.be; 11 Division of Radiology and Nuclear Medicine, Oslo University Hospital, 0027 Oslo, Norway

**Keywords:** cardiac magnetic resonance, cardiac resynchronization therapy, scar, dyssynchrony, septal flash, myocardial work

## Abstract

*Background*: The response to cardiac resynchronization therapy (CRT) depends on septal viability and correction of abnormal septal motion. This study investigates if cardiac magnetic resonance (CMR) as a single modality can identify CRT responders with combined imaging of pathological septal motion (septal flash) and septal scar. *Methods*: In a prospective, multicenter, observational study of 136 CRT recipients, septal scar was assessed using late gadolinium enhancement (LGE) (*n* = 127) and septal flash visually from cine CMR sequences. The primary endpoint was CRT response, defined as ≥15% reduction in LV end-systolic volume with echocardiography after 6 months. The secondary endpoint was heart transplantation or death of any cause assessed after 39 ± 13 months. *Results*: Septal scar and septal flash were independent predictors of CRT response in multivariable analysis (both *p* < 0.001), while QRS duration and morphology were not. The combined approach of septal scar and septal flash predicted CRT response with an area under the curve of 0.86 (95% confidence interval (CI): 0.78–0.94) and was a strong predictor of long-term survival without heart transplantation (hazard ratio 0.27, 95% CI: 0.10–0.79). The accuracy of the approach was similar in the subgroup with intermediate (130–150 ms) QRS duration. The combined approach was superior to septal scar and septal flash alone (*p* < 0.01). *Conclusions:* The combined assessment of septal scar and septal flash using CMR as a single-image modality identifies CRT responders with high accuracy and predicts long-term survival.

## 1. Introduction

Cardiac resynchronization therapy (CRT) improves left ventricular (LV) function and reduces mortality and morbidity in selected patients with dyssynchronous heart failure [1,2]. Current guidelines advocate CRT in patients with symptomatic heart failure, LV ejection fraction (EF) ≤ 35% and QRS duration ≥ 130 ms, preferably with left bundle branch block (LBBB) morphology [3]. However, QRS duration, which reflects electrical dyssynchrony, is only a moderate predictor of the CRT response, and adherence to the guideline criteria results in a non-responder rate of about 30% [4]. Therefore, better predictive tools are needed.

Patients with LBBB typically have reduced function of the interventricular septum, which directly impairs global LV function, and results in compensatory increased work of the LV lateral wall [5,6]. Such LV electromechanical dyssynchrony appears to be a substrate for CRT, and restoring septal function with CRT improves global LV performance [5]. Studies have confirmed that echocardiographic dyssynchrony indices, such as septal flash and myocardial work distribution, are associated with beneficial responses to CRT [7,8,9]. Contrarily, myocardial scar, particularly if located in the LV posterolateral wall or septum, reduces the response rate [10,11,12]. Septal viability is essential, considering the recovery of septal function a main mechanism of the CRT response [5]. In line with these findings, we recently showed that asymmetric LV work distribution with echocardiography combined with septal scar with cardiac magnetic resonance (CMR) identifies CRT responders better than the criteria recommended in current guidelines [13]. In patients with suboptimal echocardiographic images, however, alternative approaches for LV dyssynchrony imaging are needed. Unlike echocardiography, CMR image quality is independent of the acoustic window. Furthermore, CMR is needed to assess septal scar [14]. We hypothesized that imaging of LV dyssynchrony and myocardial work as well as septal scar with CMR may be used to identify patients who are likely to respond positively to CRT.

Thus, in a prospective, multicenter, observational study, we investigate if CMR as a single modality for imaging LV dyssynchrony, myocardial work and myocardial scar identifies CRT responders beyond the selection criteria recommended in current guidelines.

## 2. Methods

### 2.1. Study Population

The present study is part of a prospective, multicenter, observational study previously reported [13]. Of the total 236 patients originally enrolled, all patients who successfully completed a CMR scan and had available follow-up data were consecutively included in the present study (*n* = 136). The main reason for not performing CMR was a previously implanted CMR-incompatible cardiac device (*n* = 53). For the remaining patients (*n* = 30), the reasons included patient refusal, intracranial metal implants and logistical causes/no available CMR slot. Additionally, 3 patients were excluded due to inadequate CMR image quality. Reasons for no available follow-up data included CRT not implanted (*n* = 11), loss to follow-up (*n* = 1), lead extraction because of endocarditis (*n* = 1) and no follow-up echocardiography (*n* = 1).

The study inclusion criteria were an indication for CRT according to European Society of Cardiology (ESC) guidelines [3]. This study was conducted following the ethical guidelines of the Declaration of Helsinki and was approved by the Regional Ethical Committees of every participating center. Informed consent was obtained from each patient. This study was registered at clinicaltrials.gov (identifier NCT02525185). The present study is an exploratory observational outcome study.

### 2.2. Cardiac Magnetic Resonance (CMR)

CMR scans were performed before CRT was implanted with a 1.5 (*n* = 92) or 3.0 (*n* = 44) Tesla unit (Aera, Skyra or Verio, Siemens, Erlangen, Germany, or Ingenia, Philips Healthcare, Best, The Netherlands, or Signa HDXT, GE, Boston, MA, USA). The acquisition was performed with ECG gating and during breath-holds. Standard long- and short-axis cine sequences covering the entire left ventricle were acquired using a steady-state free precession sequence with 31 ± 3 frames per heart cycle. LV volumes were measured, and EF was calculated from short-axis slices with the freely available software Segment v2.0 R5270 (Medviso AB, Lund, Sweden) [15]. All CMR analyses were performed at the same center.

#### Myocardial Scar

We performed late gadolinium enhancement (LGE) to define myocardial scar in individuals with preserved renal function (eGFR ≥ 45 mL/min/1.73 m^2^). Images in long- and short-axis projections were obtained during steady-state after intravenous injection of either 0.15 (*n* = 79) or 0.20 (*n* = 44) mmol/kg gadoterate meglumine (Doteram™, Guerbet, Villepinte, France), 0.15 mml/kg gadobutrol (Gadovist™, Bayer AB, Stockholm, Sweden) (*n* = 3) or 0.15 mmol/kg gadobenate dimeglumine (MultiHance^®^, Bracco, Milan, Italy) (*n* = 2). A trained CMR radiologist assessed LGE visually and defined etiology as ischemic or not. From a stack of short-axis slices, LGE volume was quantified semi-automatically with Segment software v2.0 R5270 and reported with percentage LGE per associated tissue volume in a 17-segment model. We utilized the automatic algorithm EWA (expectation maximization, weighted intensity, a priori information) [16]. Quantitative analysis using the semi-automatic software was made independently of the visual analysis, and any discrepancies between the two were addressed and conclusions were made between readers.

Reduced renal function precluded contrast agent administration in 8 of 136 patients. Patients available for LGE analysis included the 125 patients previously reported [13], and 3 patients with incomplete echocardiographic strain data. In one patient, image artifacts from an implantable cardioverter defibrillator led us to exclude the LGE analysis in the anterior wall and septum. Hence, the total number of patients available for analyses involving septal LGE was 127.

### 2.3. Indices of LV Dyssynchrony

#### 2.3.1. Septal Flash

**Figure 1:**
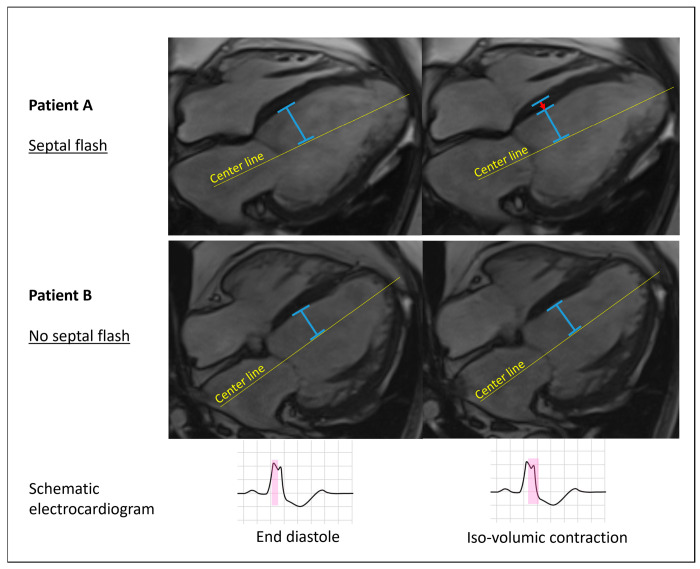
Schematic illustration of septal flash with cine 4-chamber images. The two images per patient are sequential phases of the 4-chamber cine-CMR images. The yellow longitudinal line is in a similar position and has the same length in both images from each patient. The short, transverse blue line marks the endocardial contour of the septum. Patient A displays an early contractile motion of the interventricular septum (septal flash) indicated by the red arrow, with no apparent motion of the lateral wall during the iso-volumic contraction phase (second image). Patient B demonstrates no pre-ejection septal movement (no septal flash). The pink color in the schematic electrocardiogram at the bottom of the figure shows the timing of events in the cardiac cycle.

Septal flash [7] was defined as an early and fast left–right motion (thickening/thinning) of the interventricular septum that starts and mostly ends during the isovolumic contraction phase prior to aortic valve opening (Figure 1 and Video S1). It was determined to be present if visualized in 4-chamber long-axis or any of the short-axis cine images as a yes or no phenomenon. To test reproducibility of septal flash, we performed intercenter variability testing in 25 randomly selected patients. Septal flash was assessed in all 136 patients.

#### 2.3.2. Myocardial Work

Septal and lateral myocardial work was calculated with an LV pressure–strain analysis, as previously described [17], using circumferential strain from feature tracking software (2D CPA MR v2.7.2; TomTec Imaging Systems, Unterschleissheim, Germany) and non-invasive LV pressure [18]. Systolic shortening is positive work, while lengthening is negative work. Net work is the sum of positive and negative work. The lateral-to-septal work difference was calculated as the absolute difference in net work performed by the LV lateral wall and septum and used as a marker of dyssynchronous LV workload. Blood pressure was measured using the brachial cuff method on the same day as the CMR examination in an equivalent resting condition. Myocardial work was assessed in 130 of 136 patients. In the remaining six patients, image quality was insufficient for strain analysis.

### 2.4. Echocardiography

All patients underwent echocardiographic examination (Vivid E9 or E95, GE Vingmed Ultrasound, Horten, Norway) at baseline and 6 (6 ± 1) months follow-up. LV volumes were calculated using the biplane Simpson’s method using two-dimensional images from the apical views. Septal flash was assessed with similar criteria as CMR, as previously reported [13].

### 2.5. Device Implantation

A biventricular system was implanted according to standardized directions. Coronary venography was used to delineate venous anatomy, and the LV lead was placed in a lateral or posterolateral vein, if possible. The device was programmed in a conventional biventricular pacing modus and tested to ensure it was technically well-functioning prior to hospital discharge.

### 2.6. Endpoints

Reverse remodeling is closely related to mortality, and a ≥15% reduction in LV end-systolic volume (ESV) with echocardiography at 6 (6 ± 1) months follow-up compared with baseline was defined as the primary endpoint [19]. Three different centers measured all volumes independently. In case of disagreement on response between readers, we used averaged volumes from the two agreeing readers.

The secondary endpoint was heart transplantation or death of any cause 39 ± 13 months after device implantation.

### 2.7. Statistical Analyses

Continuous variables are expressed as mean ± SD if normally distributed; otherwise, continuous variables are expressed as the median (interquartile range). The Student’s t-test, Mann–Whitney U test or chi-square test were used, as appropriate, to compare groups. To identify significant predictors for reverse remodeling (primary endpoint), we used linear regression analysis with left ventricular end-systolic volume change as a dependent continuous variable. The variance inflation factor (VIF) was used as a measure of multicollinearity, and VIF < 5 was considered acceptable for inclusion in multivariable regression analysis.

Receiver operating characteristic (ROC) curves with area under the curve (AUC) and 95% confidence intervals (CIs) were used to determine discriminative ability. To assess the discriminative ability of two parameters combined, we used logistic regression to calculate a linear combination of the parameters, which was then used for ROC curves. The Hanley and McNeil method [20] was used to compare ROC curves.

To assess long-term survival (secondary endpoint), we used a hazard ratio with a 95% CI from Cox regression and a log-rank test from Kaplan–Meier curves. Censoring was administrative due to individuals entering the study at different time points and, hence, different observation times. Intercenter variability in septal flash was assessed using the intraclass correlation coefficient (ICC). Statistical significance was set at a two-tailed probability level of *p* < 0.05. SPSS version 25.0 (IBM Corporation, Armonk, NY, USA) and MedCalc version 20.010 (MedCalc Software Ltd., Ostend, Belgium) were used for analyses.

## 3. Results

### 3.1. Study Population

The baseline characteristics of all patients are presented in Table 1. In the total population, 103 patients (76%) responded to CRT with reverse remodeling. In the subgroup with intermediate QRS duration (130–150 ms) (*n* = 29), the response rate was 62%. One patient received a heart transplant and two died during the 6-month follow-up: all three were considered non-responders. Responders had broader QRS complexes and were more likely to have LBBB morphology compared with non-responders (Table 1). Eighteen patients (13%) died or underwent heart transplantation during the follow-up (39 ± 13 months).

### 3.2. Septal Scar (LGE)

The median total scar burden was 0.3% (interquartile range: 0.0–10.0) in all patients and 10.0% (interquartile range 3.2–20.0) in patients with LGE. Sixty-four patients had some degree of LGE: 47 in the anterior wall, 59 in the septum, 56 in the inferior wall and 39 in the lateral wall. LGE classification and distribution is illustrated in Table 2. Several patients had LGE in more than one location, like RV insertion fibrosis, which almost always affected both septum and the inferior wall. Supplemental Figure S1 illustrates non-ischemic LGE examples and one artifact example.

In the multivariable regression analysis with LV end-systolic volume change as the dependent continuous variable, we tested percentage LGE in the four walls in addition to QRS duration and QRS morphology. Septal LGE was identified as the only significant predictor of reverse remodeling. Percentage septal LGE correlated inversely to reverse remodeling (r_s_ = −0.56, *p* < 0.001) and predicted CRT response with an AUC of 0.79 (95% CI: 0.70–0.89). In comparison, AUC for QRS duration was 0.62 (95% CI: 0.51–0.74) and for QRS morphology 0.58 (95% CI: 0.46–0.70).

**Figure 2:**
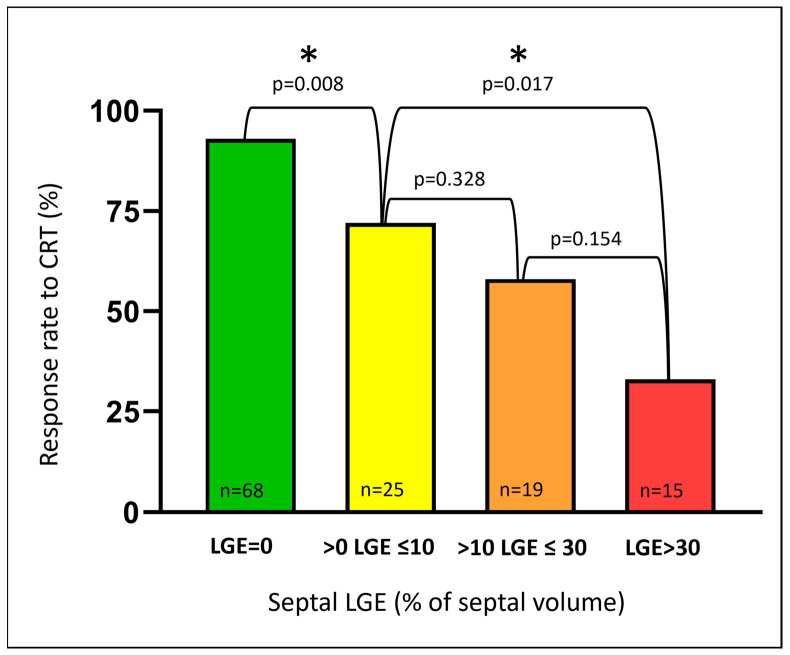
Response rates to CRT stratified to increasing amounts of septal LGE. An increasing amount of septal LGE resulted in decreasing response rates to CRT. Even minor septal LGE significantly reduced the likelihood of response compared with no septal LGE. LGE = late gadolinium enhancement; CRT = cardiac resynchronization therapy. * Denotes statistically significant differences (*p* < 0.05) between groups.

As illustrated in Figure 2, the response rate declined with an increasing amount of septal LGE. However, the mere presence of septal LGE significantly reduced the likelihood of the CRT response. With no septal LGE (*n* = 68), the response rate was excellent (93%). In comparison, the response rate was only 58% in patients with any septal LGE (*n* = 59) (*p* < 0.001, compared with no septal LGE). The response rates were similar whether septal LGE was ischemic or non-ischemic (54% vs. 67%, respectively, *p* = 0.352). LGE in other regions, not affecting the septum, did not reduce the response rate in the same way as LGE involving the septum (80% vs. 58% response).

Septal LGE was a strong predictor of long-term mortality and heart transplantation with a hazard ratio of 5.0 (95% CI: 1.8–14.4) compared with no septal LGE (*p* = 0.0026) and was the only significant predictor in multivariable analysis including age, indexed end-diastolic volume and NYHA class.

### 3.3. Septal Flash

Septal flash was more frequent in responders (Table 1). The response rate was 88% if septal flash was present, as compared with 34% if septal flash was absent (*p* < 0.001). The AUC for CRT response prediction was 0.77 (95% CI: 0.66–0.87). Septal flash was associated with improved long-term survival without heart transplantation with a hazard ratio of 0.24 (95% CI: 0.08–0.75).

Reproducibility testing revealed agreement in 24 of the 25 randomly selected patients. Intercenter ICC was 0.96 (95% CI 0.90–0.98), indicating excellent reproducibility.

### 3.4. Combining Septal LGE and Septal Flash

**Figure 3:**
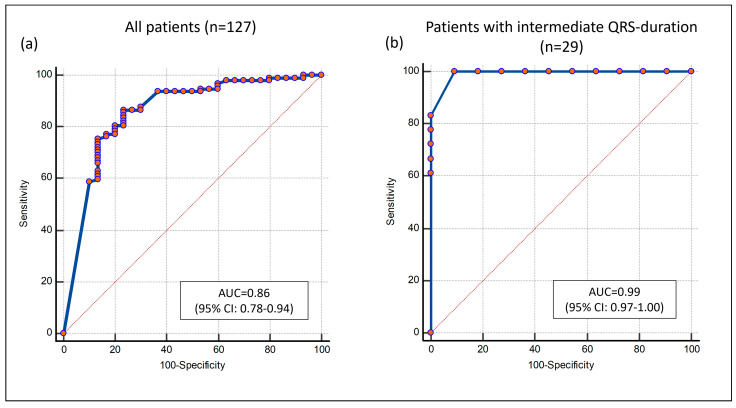
Receiver operator characteristic curve for predicting CRT response with the combined approach of percentage septal LGE and septal flash. (**a**) All available patients (*n* = 127). (**b**) The subgroup of patients with intermediate QRS duration (*n* = 29). Each point on the ROC curves represents a sensitivity/specificity pair corresponding toa particular decision threshold. CRT = cardiac resynchronization therapy; LGE = late gadolinium enhancement; AUC = area under the curve; CI = confidence interval.

In the multivariable regression analysis including percentage septal LGE, septal flash, QRS duration and QRS morphology, septal LGE and septal flash were the only significant independent predictors of reverse remodeling (Table 3). Furthermore, the percentage of septal LGE and septal flash showed an incremental value to a multivariable model for CRT response including QRS duration, QRS morphology, heart failure etiology and indexed LV ESV (both *p* < 0.01). The combined approach of percentage septal LGE and septal flash predicted CRT response with AUC = 0.86 (95% CI: 0.78–0.94). Accuracy was similar in the subgroup with intermediate QRS duration (AUC = 0.99 (95% CI 0.97–1.00)) (Figure 3).

**Figure 4:**
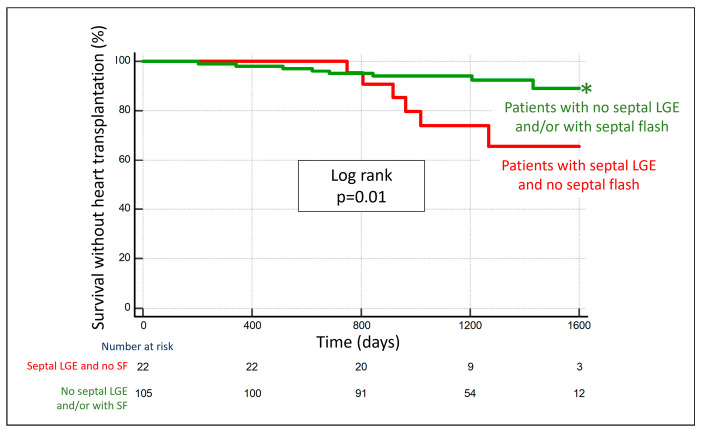
Patients without septal LGE and/or with septal flash have significantly better long-term survival as compared with patients with septal LGE and no septal flash. Kaplan–Meier curve stratified according to whether patients had (1) no septal LGE and/or with septal flash (green) or (2) septal LGE and no septal flash (red). * Denotes statistically significant (*p* < 0.05) differences between the groups. LGE = late gadolinium enhancement; SF = septal flash.

No septal LGE indicated an excellent response rate. Thus, the incremental value of septal flash was most pronounced in patients with septal LGE, where the response was more diverse. In this group, septal flash significantly improved the accuracy compared with septal LGE alone (*p* = 0.0045 for ROC curve comparison). Patients with septal LGE and septal flash had a high likelihood of response (78%), while patients with septal LGE and *no* septal flash were unlikely to respond (23%). The graphical abstract outlines an algorithm based on the two parameters, which correctly classified 86% of all patients as responders or non-responders. Importantly, the accuracy of the algorithm was similar in the subgroup of patients with intermediate QRS duration (93% of patients correctly classified). Furthermore, patients who were classified as likely responders with the algorithm had significantly better long-term survival without heart transplantation compared with patients who were classified as likely non-responders (hazard ratio of 0.27 (95% CI: 0.10–0.79) (Figure 4).

### 3.5. Lateral-to-Septal Work Difference

The difference in myocardial workload between the LV lateral wall and septum correlated to reverse remodeling (r_s_ = −0.25, *p* = 0.005). An increased work difference was associated with more reverse remodeling. Combining septal LGE and lateral-to-septal work difference predicted the CRT response with AUC = 0.84 (95% CI: 0.75–0.92). However, work difference was less suited than septal flash to distinguish responders from non-responders among patients with septal LGE, where it was not better than percentage septal LGE alone (*p* > 0.1 for comparison of ROC curves).

### 3.6. Septal Flash with Echocardiography

Septal flash with CMR agreed with septal flash with echocardiography in 121 of 133 patients (91%): Six had septal flash with CMR and not echocardiography and six had septal flash with echocardiography and not CMR. Combining septal flash with echocardiography with septal LGE with CMR yielded AUC = 0.82 (95% CI 0.73–0.92) for CRT response prediction, which was similar to the equivalent combination with CMR as a single modality (*p* = 0.22).

## 4. Discussion

The novel finding of the present prospective, multicenter study is that combined assessment of septal scar (LGE) and septal flash with CMR as a single image modality identifies CRT responders with high accuracy and predicts long-term survival. We suggest a simple algorithm based on these two parameters, which predicts CRT response beyond current guideline criteria. With no septal LGE, the response rate to CRT is excellent irrespective of other parameters. If septal LGE is present and there is septal flash, patients are likely to respond. If, on the other hand, septal LGE is present and there is no septal flash, a response to CRT is highly unlikely. Importantly, the accuracy of the algorithm is similar in patients with intermediate QRS duration. In this group, the method may be particularly useful because the benefit of CRT is less consistent.

The suggested approach, unlike many previously reported methods [3], does not require multimodality imaging. It is available for patients where poor image quality precludes echocardiographic evaluation and is quick and easy to perform. Therefore, it is a clinically attractive improvement to patient selection for CRT.

### 4.1. Septal Markers Define the CRT Response

LGE is a marker of adverse structural remodeling in response to myocardial injury and increased wall stress, and the extent of LGE varies in patients despite similar degrees of myocardial dysfunction [21]. We found septal LGE, ischemic or non-ischemic, to be a strong predictor of non-response to CRT and adverse long-term outcome. A former study of 23 CRT recipients found that septal LGE ≤ 40% provided a 100% sensitivity and specificity for CRT response [12]. In the present larger study, however, the mere presence of septal LGE, rather than the absolute amount, is the factor that affects the response to CRT. In contrast, LGE in other regions, not involving the septum, did not seem to affect the response rate. While a transmural posterolateral wall scar is an established risk factor for a poor CRT response, the significance of lateral wall LGE per se seemed minor compared with septal LGE in the present study. A plausible explanation for this could be that most LV lateral wall scars in the present study were not transmural, which probably did not impact the efficiency of lateral wall pacing as much as with transmural scars. Due to the limited number of patients with lateral wall scars, there is need for further validation in larger populations of the impact on risk of this scar localization.

Despite inferior temporal resolution compared with echocardiography, the present study demonstrates that CMR identification of septal flash is equally clinically important. Therefore, CMR and echocardiography may be complementary image modalities in identifying LV dyssynchrony in some patients. Intercenter reproducibility of septal flash with CMR in our material was excellent. However, reproducibility might be lower if performed by less experienced readers.

Septal flash signals a potential substrate for the CRT response [7]. In line with our findings, a former smaller study by Sohal et al. also identified septal flash assessed with CMR as an independent predictor of reverse remodeling after CRT [22]. Sohal assessed septal flash by analyzing time–volume curves, while we performed a rapid visual assessment. Along the same line, Zweerink and co-workers demonstrated that systolic septal stretching, evaluated on CMR cine sequences, is a prognostic measure for good clinical outcomes after CRT [23]. The approach used in the present study has obvious clinical advantages but did not allow for the quantification of the degree of septal flash and its relation to the CRT response. In total, the results of the present study add to the growing evidence that the septum plays an essential role in transferring the harmful effects of LBBB on myocardial function and that reversing septal dysfunction constitutes a key for the CRT response. The results in the clinically challenging subgroup with intermediate QRS duration are promising, but further research is warranted due to the low patient number in this subgroup.

### 4.2. LV Lateral-to-Septal Work Difference

We previously showed, applying speckle-tracking echocardiography to measure myocardial strain, that a high LV lateral-to-septal work difference predicts the CRT response [13]. The present study demonstrates that the lateral-to-septal work difference assessed with CMR also is a predictor for the response to the treatment. Somewhat lower AUC may indicate that LV segmental strain with speckle tracking echocardiography is superior to current versions of feature tracking CMR. In patients with low-quality echocardiographic images, however, myocardial work index with CMR may have a role.

Nevertheless, in the present material, we identified septal flash as a more suited signal of unexploited contractile reserve in septum potentially recovered with CRT than the LV work difference. One possible explanation is that there may be a high difference in workload between the lateral wall and septum both in cases of pure LV electromechanical dyssynchrony (with excellent response rate to CRT) and in cases with a viable LV lateral wall and a large septal scar, and therefore no electrical substrate for CRT response. In contrast to echocardiography, CMR may characterize both septal scar and LV dyssynchrony as a single-image modality. Future larger studies should compare the clinical relevance of LV dyssynchrony assessed with CMR and echocardiography, respectively.

Despite extensive research, no imaging-based parameter of LV dyssynchrony is currently included in the CRT selection guidelines [3], largely due to the negative results of the PROSPECT trial [24]. However, most of the echocardiographic dyssynchrony indices tested as markers of the CRT response in that trial were timing indices, which are sensitive to non-electrical causes of LV dyssynchrony [25,26], where correction with CRT is unlikely [27,28]. More recent research suggests that electromechanical dyssynchrony as reflected in abnormal septal motion (septal flash) and asymmetric LV work distribution are better markers of the CRT response. Furthermore, several studies suggest that imaging of myocardial scar tissue with CMR provides added value as a predictor of the CRT response. These important functional and structural features of dyssynchronous ventricles remain to be tested in prospective studies with clinical endpoints.

Technological advances may improve the correction of dyssynchronous heart failure in the future. Recent findings suggest that novel and more physiological pacing techniques, such as left bundle branch area pacing, may be superior to conventional biventricular pacing [29] and better preserve LV function compared with right ventricular pacing in patients with pacing indication for bradycardia [30]. Implementation of left bundle branch area pacing as a potentially superior approach to bi-ventricular pacing also requires good diagnostic methods to identify patients who are likely responders. Probably, the criteria for the selection of patients will not be much different from those used for CRT, but this remains to be determined in future clinical trials.

### 4.3. Clinical Implications

The persistently high number of non-responders to CRT calls for better tools to identify responders. The present study suggests a novel and clinically attractive algorithm based on septal LGE and septal flash with CMR as a stand-alone image modality, which identified CRT responders with high accuracy and predicted long-term survival. CMR represents an additional cost compared with echocardiography but given the importance of diagnosing septal LGE in CRT candidates, we advocate increased priority for these patients to CMR. Less stringent requirements for renal function and increased use of CMR-compatible devices will probably result in more patients being eligible for CMR in the near future. Due to the lower number of responders, the proposed approach appears especially valuable for patients with intermediate QRS duration.

### 4.4. Limitations

In the present study, we did not include patients with low eGFR or patients with CMR-incompatible implants to avoid any harm inflicted upon study participants. This may have caused a selection bias. Data on LV lead position and intrinsic LV electric delay were not available, and such data might have provided additional insights. The number of transmural LV lateral wall scars was too low to investigate to a full extent. Different CMR units with different magnetic fields (1.5 or 3.0 Tesla) may have caused slight heterogeneity in the quantification of LGE, although this is not likely to affect the overall results of this study. The present study was observational, included a limited number of patients and the primary endpoint was the surrogate marker ESV reduction. Survival analysis should be interpreted with caution due to the low number of events. To change clinical practice, there is a need for a randomized trial with clinical endpoints.

## 5. Conclusions

The combined assessment of septal LGE and septal flash with CMR accurately identifies CRT responders. The accuracy is similar in the clinically relevant subgroup with intermediate QRS duration. Patients with no septal LGE have excellent response rates. In patients with septal LGE, septal flash separates responders from non-responders with high precision. Further studies are needed to verify that the novel algorithm might be used to improve patient selection for CRT.

## Figures and Tables

**Table 1:** Baseline clinical and CMR characteristics.

	All Patients (*n* = 136)	Responders (*n* = 103)	Non-Responders (*n* = 33)	*p*-Value
Age (years)	66 ± 10	67 ± 9	64 ± 11	0.071
Male sex—no. (%)	92 (68)	65 (63)	27 (82)	0.046
NYHA functional class—no. (%)				
I	6 (4)	6 (6)	0 (0)	
II	85 (63)	65 (63)	20 (61)	
III	44 (32)	32 (31)	12 (36)	
IV	1 (1)	0 (0)	1 (3)	
Medications—no. (%)				
ACE-inhibitor/ARB	131 (96)	100 (97)	31 (94)	0.403
Beta blocker	124 (91)	92 (89)	32 (97)	0.178
Aldosterone antagonist	56 (41)	42 (41)	14 (42)	0.797
Sinus rhythm—no. (%)	129 (95)	99 (96)	30 (91)	0.239
Heart failure etiology—no. (%)				
Ischemic	42 (31)	23 (22)	19 (58)	<0.001
Non-ischemic	94 (69)	80 (78)	14 (42)	<0.001
QRS duration (milliseconds)	164 ± 17	166 ± 16	158 ± 18	0.021
Left bundle branch block (%)	124 (91)	98 (95)	26 (79)	0.004
LV EDV indexed (ml/m^2^)	145 ± 46	139 ± 46	164 ± 41	0.008
LV ESV indexed (ml/m^2^)	76 ± 32	73 ± 31	88 ± 32	0.580
LV ejection fraction (%)	27 ± 8	28 ± 8	23 ± 6	0.003
Anterior LGE (%)	0 (0–6.5)	0 (0–0.1)	12.2 (0.8–36.2)	<0.001
Septal LGE (%)	0 (0–12.2)	0 (0–3.2)	16.3 (1.7–39.6)	<0.001
Inferior LGE (%)	0 (0–9.8)	0 (0–3.9)	10.5 (0.4–30.1)	<0.001
Lateral LGE (%)	0 (0–5.5)	0 (0–0)	5.6 (0–23.1)	<0.001
Septal flash—no. (%)	104 (76)	92 (89)	12 (36)	<0.001
Lateral-to-septal work difference (mmHg·%)	1551 ± 1080	1710 ± 1085	1061 ± 917	0.003

Continuous variables are given as mean ± standard deviation, except LGE in different LV regions, which is given as the median (interquartile range) because the distribution is skewed. Median LGE was zero in all LV regions for both the complete patient group and CRT responders because two-thirds of the patient population had non-ischemic cardiomyopathy. The *p*-value reports a comparison of responders vs. non-responders. CMR = cardiac magnetic resonance; NYHA = New York Heart Association; LV = left ventricular; LGE = late gadolinium enhancement.

**Table 2:** Classification and distribution of LGE.

	Number of Patients	Number with Septal Involvement (% of Total)
LGE present Ischemic Non-ischemic Combined	64 37 20 7	59 (92%) 33 (89%) 19 (95%) 6 (86%)
Location of infarcts (ischemic LGE) Anterior wall Lateral wall Inferior wall	25 14 12	25 (100%) 10 (71%) 11 (92%)
Classification of non-ischemic LGE RV insertion point fibrosis Septal midwall fibrosis Other	12 7 12	11 (92%) 7 (100%) 11 (92%)

LGE = late gadolinium enhancement.

**Table 3:** Multivariable linear regression analysis with left ventricular end-systolic volume change as a dependent continuous variable.

	Multivariable Analysis			
Regression Variable	B	95% CI	VIF	*p*-Value
QRS duration (ms)	−0.036	−0.249 to 0.177	1.158	0.738
Left bundle branch block (yes/no)	−9.18	−21.31 to 2.94	1.110	0.136
Septal LGE (%)	0.521	0.311 to 0.731	1.153	<0.001
Septal flash (yes/no)	−18.39	−27.16 to −9.61	1.325	<0.001
Constant term	−8.513			

N = 125. R^2^ = 0.40. Septal LGE is given as a continuous variable (%). B = unstandardized regression coefficient; CI = confidence interval; VIF = variance inflation factor; LGE = late gadolinium enhancement.

## Data Availability

The datasets used and analysed during the current study are available from the corresponding author upon reasonable request.

## Supplementary Materials

The following supporting information can be downloaded at: https://www.mdpi.com/article/10.3390/jcm12227182/s1, Figure S1. Examples of non-ischemic LGE; Video S1: Septal flash.
