# Supplementary material for: Cardiac Magnetic Resonance Identifies Responders to Cardiac Resynchronization Therapy with an Assessment of Septal Scar and Left Ventricular Dyssynchrony
Source: J Clin Med. 2023 Nov 20;12(22):7182. doi: 10.3390/jcm12227182 (PMC10672328; doi:10.3390/jcm12227182)
Supplement: Supplementary file 1 [file jcm-12-07182-s001.zip › Legends Figure S1and Video S1.pdf]

Supplemental material

Figure S1. Examples of non-ischemic LGE.

A): Example of midwall fibrosis (LGE) located in septum. B): Illustration of fibrosis (LGE) in the RV insertion point. C): Apparent hyperenhancement in the most basal short-axis slice, classified as partial volume effect, and not LGE.

LGE=late gadolinium enhancement; RV=right ventricular

Video S1. Septal flash.

The video displays the rapid, early movement of the interventricular septum (septal flash) in 4-chamber view in a representative patient.
